# Therapeutic Drug Monitoring to Optimize Risperidone Treatment in Children with Autism Spectrum Disorder

**DOI:** 10.1097/FTD.0000000000001161

**Published:** 2023-11-27

**Authors:** Rebecca A. Hermans, Alaya E.M. Storm, Sanne M. Kloosterboer, Manon H.J. Hillegers, Birgit C.P. Koch, Bram Dierckx, Brenda C.M. de Winter

**Affiliations:** * Department of Child and Adolescent Psychiatry/Psychology, Erasmus University Medical Center, Rotterdam, the Netherlands;; † Department of Hospital Pharmacy, Erasmus University Medical Center, Rotterdam, The Netherlands; and; ‡ Rotterdam Clinical Pharmacometrics Group, Erasmus University Medical Center, Rotterdam, the Netherlands.

**Keywords:** antipsychotic, risperidone, therapeutic drug monitoring, pharmacometrics, pediatric population

## Abstract

**Background:**

Risperidone is an atypical antipsychotic drug used to treat irritability and aggression in children and adolescents with autism spectrum disorder. In an earlier study, the sum trough concentration of risperidone and its metabolite (9-hydroxyrisperidone) was positively correlated with weight gain and effectiveness. The aim of this study was to determine the therapeutic window for risperidone sum trough concentrations that balances weight gain with treatment effectiveness in this population. In addition, the effect of therapeutic drug monitoring (TDM) on treatment optimization was simulated.

**Methods:**

In a retrospective cohort (n = 24 children), the target window for risperidone leading to the least increase in body mass index z-scores while retaining effectiveness as measured by the irritability subscale of the Aberrant Behavior Checklist was determined using receiver operating curve analysis. This target range was used to simulate the effect of TDM using a population PK model implemented in the software platform InsightRX. Dosing advice was based on plasma trough concentrations and the dose administered at 12 weeks to simulate whether more children would be on target at 24 weeks after the start of treatment.

**Results:**

A risperidone sum trough target range of 3.5–7.0 mcg/L would minimize increase in body mass index z-score and optimize effectiveness. Dosing advice using TDM and a population PK model would lead to a larger proportion of children achieving the target concentration range (62.5% versus 16.7%).

**Conclusions:**

TDM may be a useful tool for optimizing risperidone treatment in children and adolescents with autism spectrum disorder.

## INTRODUCTION

Autism spectrum disorder (ASD) is a developmental disorder affecting just below 1% of the world's population.^1^ ASD is characterized by problems in social communication and social interaction and restricted, repetitive sensory-motor behavior.^2^ Around 20% of people with ASD also experience irritability and aggression, which seriously affect their interactions with family and others, the implementation of therapies, and long-term outcomes.^3,4^ Irritability and aggressive behavior can be treated with medication, although options are limited, because only risperidone and aripiprazole are approved by the Food and Drug Administration. Risperidone and aripiprazole can be prescribed to children with ASD from the age of 5. Park et al^5^ found that 1 in 9 children and adolescents with ASD were treated with risperidone and multiple studies have proven its effectiveness in adults and children with ASD.^6–8^

The trade-off for risperidone use in children and adolescents includes adverse effects that can seriously affect physical health. The side effects include weight gain and metabolic abnormalities such as hypertension and dyslipidemia, a higher chance of developing diabetes mellitus type 2, and prolactin elevation.^9–11^ Several studies have found that the risk of these side effects increases with a higher dose of risperidone.^12,13^

Kloosterboer et al^14^ were the first to investigate the relationship between the concentrations of risperidone and its metabolite 9-hydroxyrisperidone and weight gain and effectiveness in children and adolescents with ASD. They found a positive relationship between the sum (risperidone + 9-hydroxyrisperidone) trough concentrations and increasing body mass index (BMI) z-score and more effectiveness measured using the Aberrant Behavior Checklist irritability (ABC-I) score. It may therefore be possible to use therapeutic drug monitoring (TDM) to optimize treatment in this population.^15^

In this study, as a precursor to a large randomized trial, we first determined the therapeutic reference range for the sum trough concentration of risperidone in this population. Second, using a population PK model, we simulated whether changes in the risperidone dose based on TDM would lead to more children reaching this target concentration.

## MATERIALS AND METHODS

### Participants

This study used subject data from Kloosterboer et al,^14^ which consisted of 42 children and adolescents aged 5–17 years who were diagnosed with ASD. For the analysis of the therapeutic range, participants were excluded if they had started using risperidone before the beginning of the study or if the necessary outcome measurements were missing. For the simulations, participants were excluded if they had started using risperidone before the beginning of the study, did not complete 24 weeks of risperidone use, or were taking more than 2 risperidone doses per day. Furthermore, plasma concentration measurements were excluded if the dose or time of medication intake was unclear. Finally, participants were excluded if they did not have (remaining) plasma concentration measurements at weeks 12 or 24.

### Materials

#### Risperidone Concentrations

Blood samples were collected 12 and 24 weeks after the start of risperidone treatment using venipuncture or the dried blood spot (DBS) method. Samples were collected at random time points after dose administration. DBS is a less invasive and more child friendly way to quantify drug concentrations for pharmacokinetic studies (Patel et al, 2010).^16,17^ DBS concentrations were converted using formulas with correction for hematocrit (ht) into estimated plasma concentrations: EPC_risperidone_ = (DBS_conc_/(1−ht))/1.120, and EPC_9−hydroxyrisperidone_ = (DBS_conc_/(1−ht))/0.996.^18^ The lower limit of quantification was 1 mcg/L for risperidone and 0.7 mcg/L for 9-hydroxyrisperidone, both for DBS and venipuncture. The lower limit of detection (LOD) for risperidone was 0.02 mcg/L in plasma and 0.9 mcg/L in DBS. The LOD for 9-hydroxyrisperidone was 0.22 mcg/L in plasma and 0.5 mcg/L in DBS.

#### Therapeutic Range

The target range was determined using data from Kloosterboer et al^14^ and based on the sum trough concentrations at 12 weeks after the start of therapy as predicted by the developed PK model, change in BMI between baseline and 24 weeks after start of treatment, and change in symptom severity between baseline and 12–24 weeks after start of treatment. BMI values were adjusted for age and weight and transformed into BMI z-scores based on the reference values (5–19 years) of the World Health Organization.^19^ The ABC-I score, which is accepted to be the gold standard for measuring these symptoms in ASD medication trials, was used to measure symptom severity.^20,21^

Receiver operating curve (ROC) analyses were performed to determine the cutoff values. For BMI z-score, an increase of 0.5 or higher was considered as significant weight gain. Treatment was considered effective if the ABC-I score decreased by 25% or more. If the BMI z-score was missing at 24 weeks but known at 12 weeks, the last observation carried forward principle was applied.

#### Pharmacokinetic Analyses

In this study, the PK model for risperidone and 9-hydroxyrisperidone by Kloosterboer et al was implemented in InsightRX (version 1.39.7, San Francisio, CA) as the user interface.^14,22^ An important difference between the model published by Kloosterboer et al and the model in InsightRX is the absence of additional and proportional errors for the lower limit of quantification and LOD in the InsightRX model. Because of this difference, we decided to exclude plasma concentration measurements below the LOD when the concentration predicted by InsightRX at the time of sampling was also lower than the LOD. When the estimation was higher than the LOD, a concentration value half the LOD was used. Furthermore, the data from InsightRX cannot be automatically extracted. Instead, the data were extracted manually, and data implementation and extraction were evaluated by a second person.

Participant data (date of birth, data concerning risperidone concentration measurements, prescribed doses, and weight at 12 and 24 weeks after the start) were entered into InsightRX. First, only the data at 12 weeks were fed into Insight RX. The middle of the target concentration range was used as the target value. The dose suggested by InsightRX (dose_sim_), for which the predicted target sum trough concentration (C_target_) was closest to the target value, was chosen and rounded to 0.05 mg. The dosing interval did not change during TDM dosing.

Second, data from 24 weeks were entered, and the prescribed dose that the children received per day at 24 weeks (dose_obs_) and the accompanying sum trough concentration at 24 weeks (C_obs_) were extracted. Because blood samples were collected using random sampling, the sum of the trough concentrations had to be simulated using InsightRX. The sum trough concentrations were extracted when steady-state was reached before the first dose of the day at 24 weeks. The simulated sum trough concentration (C_sim_) at 24 weeks, when the child would have taken the dose_sim_, was calculated using the following formula:

$$C_{\text{sim}}=\left({\text{dose}}_{\text{sim}}*C_{\text{obs}}\right)/{\text{dose}}_{\text{obs}}.$$

C_sim_ was then compared with C_target_ and the number of children who ended up in the target range after dose_sim_ (simulated) was compared with that after dose_obs_ (observed).

### Statistical Analyses

ROC analysis was performed using SPSS software (version 28; IBM, Armonk, NY). All other statistical analyses were performed using the R Studio software (version 3.4.1, R Foundation for Statistical Computing, Vienna, Austria). Figures were created using SPSS software. A paired *t* test was used to compare C_target_ and C_sim_ at 24 weeks to determine whether the dosing advice would lead to the target concentration at 24 weeks. To test the differences in the proportion of children within, below, and above the target range after dose_sim_ and dose_obs_, a two-sample test for equality of proportions with continuity correction was performed. Continuous variables are described as median [interquartile range (IQR)] and proportions as percentages (95% CI). Nonparametric alternatives were used when the assumptions were not met.

## RESULTS

### Therapeutic Range

#### Participants

Eleven children were excluded because they had started using risperidone before the beginning of the study, and 1 child was excluded because there was no known sum trough concentration at 12 weeks. The remaining 30 children were included in the analysis of the upper cutoff value. Owing to missing ABC-I scores, only 26 children were included in the analysis of the lower cutoff value. Table 1 shows the baseline characteristics of the children included in the analysis.

**TABLE 1.** Relevant Baseline Characteristics Per Analysis

Characteristic	Analysis of Upper Cut-Off (n = 30)	Analysis of Lower Cut-Off (n = 26)	Simulation Study (n = 24)
Male (%)*	23 (76.7)	20 (76.9)	19 (79.2)
Age† (yr)	10.4 (8.5–13.8)	10.4 (8.6–13.4)	10.7 (8.5–13.8)
Height† (cm)	143 (131–166)	143 (132–156)	146 (132–166)
Weight† (kg)	33.7 (26.2–46.7)	33.7 (26.5–44.1)	38.1 (26.5–46.7)
BMI z-score†	−0.26 (−0.90 to 0.65)	—	—
ABC-I score†	—	12 (4–25)	—
Dose week 12† (mg)	0.50 (0.50–1.00)	0.50 (0.50–1.00)	0.50 (0.50–1.00)
Sum C_trough_ week 12† (µg/L)	7.87 (3.28–10.07)	7.87 (3.28–10.83)	6.77 (3.28–10.83)

ABC-I, irritability subscale of the Aberrant Behavior Checklist.

* Presented as number of cases (%) for categorical variables.

† Presented as median (IQR) for continuous variables.

#### ROC Analysis

**FIGURE 1.**
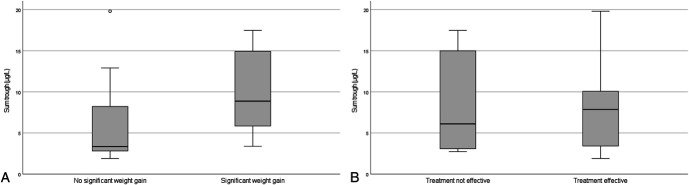
Boxplots of risperidone sum trough concentrations after 12 weeks of treatment in (A) patients with and without significant weight gain after 24 weeks of treatment and (B) patients with and without significant treatment effect after 24 weeks of treatment.

The area under the curve of the ROC analysis for the BMI z-score was 0.801. Using the Youden index, the optimal upper cutoff value was a risperidone sum trough concentration of 8.46 mcg/L. Figure 1A shows the sum trough concentrations in participants with and without significant weight gain. The area under the curve of the ROC analysis for the ABC-I score was 0.427. This means that this method cannot be used to determine the lower cutoff value. Instead, we diverted to the method to come to a preliminary therapeutic reference range described in the Consensus guidelines for TDM in neuropsychopharmacology, which is to calculate the arithmetic mean ± SD of drug concentrations of responders.^23^ A Q–Q plot and Shapiro-Wilk test (W(18) = 0.913, *P* = 0.098) showed that the trough concentrations of responders were normally distributed, and this method could thus be followed. The mean (SD) risperidone sum trough concentration of all responders was 7.77 (±4.98) mcg/L, meaning the preliminary lower cutoff value would be 2.79 mcg/L. Figure 1B shows the sum trough concentrations of participants with and without treatment response. As both cutoff values should be interpreted with caution, we decided to narrow our therapeutic range to risperidone sum trough concentrations between 3.5 and 7.0 mcg/L. As the target value for the PK analysis, the middle of this range (5.25 mcg/L) was used.

### Simulation Study

#### Participants

For the simulations, 6 more children were excluded: 2 children because of insufficient treatment time with risperidone, 3 children because they did not have (remaining) plasma concentration measurements at weeks 12 or 24, and 1 child because of a regimen of more than 2 risperidone doses per day. Of the 24 children included in the final analyses, 19 were male. The median age was 10.7 years (IQR 8.5–13.8; Table 1).

#### Pharmacokinetic Analysis

There was no significant difference between C_target_, which had a median value of 5.25 mcg/L (IQR 5.10–5.40) and C_sim_, with a median value of 5.39 mcg/L (IQR 4.56–7.02) at 24 weeks (*P* = 0.147), showing that the model accurately predicted the target concentration at 24 weeks based on the dosing advice at 12 weeks.

**FIGURE 2.**
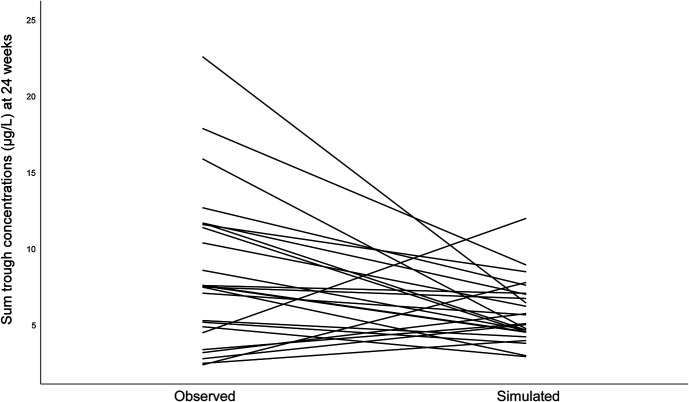
Spaghetti plot of sum trough concentrations (mcg/L) at 24 weeks after the start of the study, comparing observed and simulated data. Each line represents a single patient.

The simulated and observed dose and concentration data for 24 weeks are presented in Table 2. The difference in the sum trough concentration between the observed and simulated values at 24 weeks is schematically shown in Figure 2. Median dose_sim_ was 0.43 mg (IQR 0.20–0.60) a day. The intervention resulted in a significant decrease in dose (*P* = 0.014) and sum trough concentration (*P* = 0.023) compared with dose_obs_ (0.65 mg, IQR 0.50–1.00) with a median sum trough concentration of 7.55 mcg/L (IQR 4.80–11.60).

**TABLE 2.** Observed Versus Simulated Dose and Concentration at 24 weeks

Outcome Variable	Observed	Simulated	*P*
Dose* (mg)	0.65 (0.50–1.00)	0.43 (0.20–0.60)	0.014†
Concentration* (µg/L)	7.55 (4.50–11.60)	5.39 (4.56–7.02)	0.023†
N in therapeutic range (3.5–7 µg/L)‡	4 (16.7%)	15 (62.5%)	0.003§
N below therapeutic range (<3.5 µg/L)‡	5 (20.8%)	2 (8.3%)	0.413
N above therapeutic range (>7 µg/L)‡	15 (62.5%)	7 (29.2%)	0.043†

* Presented as median (IQR) for continuous variables.

† *P* < .05.

‡ Presented as number of cases (%) for categorical variables.

§ *P* < .01.

Results showed that more children reached the target range (3.5–7.0 mcg/L) after dose_sim_ (62.5%, 95% CI, 40.6–81.2) compared with dose_obs_ (16.7%, 95% CI, 4.7–37.4), χ^2^(1) = 8.71, *P* = 0.003 (95% CI, 0.17–0.74). Dose_sim_ also led to fewer children reaching a sum trough concentration above the target concentration range (29.2%, 95% CI, 12.6–51.1) compared with dose_obs_ (62.5%, 95% CI, 40.6–81.2), χ^2^(1) = 4.11, *P* = 0.043 (95% CI, −0.64 to −0.03). There was no difference in proportions of children with a sum trough concentration below the target range between the simulated data (8.3%, 95% CI, 10.3–27.0) and the observed data (20.8%, 95% CI, 7.1–42.2), χ^2^(1) = 0.67, *P* = 0.413 (95% CI, −0.36 to 0.11).

## DISCUSSION

In this study, we determined a target range for risperidone plasma trough concentrations in children and adolescents with ASD and examined the extent to which TDM could lead to children reaching concentrations within this target range.

We propose a target sum trough range of 3.5–7.0 mcg/L for optimal effectiveness with the least amount of side effects. Children with ASD with risperidone sum trough concentrations within this range are likely to achieve a reduction of 25% or more on the ABC-I score, without gaining more weight than would lead to an increase of 0.5 or more in BMI z-score.

To the best of our knowledge, 2 other preliminary therapeutic reference ranges for risperidone in children and adolescents have been calculated. Klampfl et al^24^ suggested a range of 8–26 mcg/L for impulsive-aggressive symptoms in a sample with 74% disruptive behavior disorders. Their lower cutoff was based on the mean serum concentration of children treated within the recommended dose range, whereas their upper cutoff was the highest concentration in the group of children without “severely impairing” side effects as rated on the UKU side effect rating scale, on which weight gain is an item. Using the arithmetic mean ± SD method with their sample would lead to a reference range that is closer to ours. Furthermore, they also reported a tendency of worse outcomes with higher concentrations (mean ± SD: 19.0 ± 20.5 mcg/L), likely reflecting the tendency to increase dosage in nonresponders, to no avail. Taurines et al^25^ suggested a reference range of 9–33 mcg/L for children and adolescents with psychotic disorders, based on treatment effectiveness and extrapyramidal side effects. It is very well possible that different cutoffs exist for different treatment indications and for different side effects. Although weight gain was included in the study by Klampfl et al, the treatment duration varied greatly within the sample, causing weight gain to be more difficult to quantify. In the original article on our data by Kloosterboer et al,^14^ a theoretical therapeutic window (15–25 mcg/L) was also given, but this was for a child with specific characteristics and based on absolute values of BMI z-score and ABC-I rather than changes in these parameters.

Our results suggest that the dose recommended by InsightRX based on plasma concentrations at 12 weeks accurately led to the target concentration at 24 weeks. This validates the InsightRX model as a useful tool for accurate dosing advice, leading to a predetermined target concentration. Furthermore, this dosing advice led to a larger proportion of children reaching the target range compared with when the dose was determined by a physician. After the implementation of TDM, the dose and sum trough concentrations at 24 weeks were significantly lower than those after the prescribed dose.

Because the risperidone dose and the sum trough concentration were lower in the simulated data, TDM would not only lead to weight loss, but also decreased effectiveness because of the positive relationship between dose and sum trough concentration and weight gain and effectiveness.^12–14^ However, concentrations within the target range should lead to an equilibrium between the least amount of weight gain and continued effectiveness.^14^

This study has some limitations. First, the therapeutic window is based on a small dataset. To account for this, we decided to narrow the window slightly to lower the chances of dosing too low or too high. Second, the simulation study only simulated the implementation of TDM at 12 weeks after start because of the data available from Kloosterboer et al.^14^ Because risperidone-induced weight gain occurs mostly during the first 15 weeks of risperidone use,^26^ it would be preferable to give dosing advice as early as possible to optimize treatment outcome. Third, the observed sum trough concentrations at 24 weeks were extracted from InsightRX based on individualized concentration curves. This method was chosen because plasma concentrations were almost never measured immediately before the next dose was administered; therefore, the actual sum trough concentrations were missing. This was deemed sufficient because the PK model was constructed using data from the same participants; thus, the model accurately described the PK of these children.

Finally, this study only used participant data from Kloosterboer et al,^14^ and the model was based on the same participants. This means that generalization to other populations and applications should be performed with caution. To improve this simulation study, data from participants in different studies should be included to make the study more generalizable and increase the sample size. Furthermore, including more time points at which plasma concentrations are measured would provide further insight into the optimal time to implement TDM.

The next step after this study is to use TDM in a prospective randomized controlled trial (RCT) to determine how our results translate into clinical practice. To this end, we have set up the SPACe 2: STAR trial.^27^ This RCT will serve as external validation of our therapeutic window, and if results from this simulation study are reproduced during the RCT, it would endorse the value of simulation studies such as this one.

## CONCLUSIONS

This study showed promising results for the implementation of TDM in children and adolescents with ASD to achieve a target concentration that would lower risperidone-induced weight gain.

## References

[1] Simonoff E Pickles A Charman T . Psychiatric disorders in children with autism spectrum disorders: prevalence, comorbidity, and associated factors in a population-derived sample. J Am Acad Child Adolesc Psychiatry. 2008;47:921–929. 18645422 10.1097/CHI.0b013e318179964f

[2] American Psychiatric Association. Diagnostic and Statistical Manual of Mental Disorders. 5th ed. Washington, DC: American Psychiatric Association; 2013.

[3] Lecavalier L. Behavioral and emotional problems in young people with pervasive developmental disorders: relative prevalence, effects of subject characteristics, and empirical classification. J Autism Dev Disord. 2006;36:1101–1114. 16897387 10.1007/s10803-006-0147-5

[4] Samson AC Phillips JM Parker KJ . Emotion dysregulation and the core features of autism spectrum disorder. J Autism Dev Disord. 2014;44:1766–1772. 24362795 10.1007/s10803-013-2022-5

[5] Park SY Cervesi C Galling B . Antipsychotic use trends in youth with autism spectrum disorder and/or intellectual disability: a meta-analysis. J Am Acad Child Adolesc Psychiatry. 2016;55:456–468 e4. 27238064 10.1016/j.jaac.2016.03.012

[6] Fallah MS Shaikh MR Neupane B . Atypical antipsychotics for irritability in pediatric autism: a systematic review and network meta-analysis. J Child Adolesc Psychopharmacol. 2019;29:168–180. 30707602 10.1089/cap.2018.0115

[7] McDougle CJ Holmes JP Carlson DC . A double-blind, placebo-controlled study of risperidone in adults with autistic disorder and other pervasive developmental disorders. Arch Gen Psychiatry. 1998;55:633–641. 9672054 10.1001/archpsyc.55.7.633

[8] Pandina GJ Bossie CA Youssef E . Risperidone improves behavioral symptoms in children with autism in a randomized, double-blind, placebo-controlled trial. J Autism Dev Disord. 2007;37:367–373. 17019624 10.1007/s10803-006-0234-7

[9] De Hert M Dobbelaere M Sheridan EM . Metabolic and endocrine adverse effects of second-generation antipsychotics in children and adolescents: a systematic review of randomized, placebo controlled trials and guidelines for clinical practice. Eur Psychiatry. 2011;26:144–158. 21295450 10.1016/j.eurpsy.2010.09.011

[10] Libowitz MR Nurmi EL. The burden of antipsychotic-induced weight gain and metabolic syndrome in children. Front Psychiatry. 2021;12:623681. 33776816 10.3389/fpsyt.2021.623681 PMC7994286

[11] Srisawasdi P Vanwong N Hongkaew Y . Impact of risperidone on leptin and insulin in children and adolescents with autistic spectrum disorders. Clin Biochem. 2017;50:678–685. 28167244 10.1016/j.clinbiochem.2017.02.003

[12] Bobo WV Cooper WO Stein CM . Antipsychotics and the risk of type 2 diabetes mellitus in children and youth. JAMA Psychiatry. 2013;70:1067–1075. 23965896 10.1001/jamapsychiatry.2013.2053

[13] Hoekstra PJ Troost PW Lahuis BE . Risperidone-induced weight gain in referred children with autism spectrum disorders is associated with a common polymorphism in the 5-hydroxytryptamine 2C receptor gene. J Child Adolesc Psychopharmacol. 2010;20:473–477. 21186965 10.1089/cap.2009.0071 PMC3003450

[14] Kloosterboer SM de Winter BCM Reichart CG . Risperidone plasma concentrations are associated with side effects and effectiveness in children and adolescents with autism spectrum disorder. Br J Clin Pharmacol. 2021;87:1069–1081. 32643213 10.1111/bcp.14465 PMC9328651

[15] Kang JS Lee MH. Overview of therapeutic drug monitoring. Korean J Intern Med. 2009;24:1–10. 19270474 10.3904/kjim.2009.24.1.1 PMC2687654

[16] Kloosterboer SM van Eijk E van Dijk M . Feasibility of dried blood spots in children with behavioral problems. Ther Drug Monit. 2020;42:648–651. 32453305 10.1097/FTD.0000000000000776

[17] Patel P Mulla H Tanna S . Facilitating pharmacokinetic studies in children: a new use of dried blood spots. Arch Dis Child. 2010;95:484–487. 20501544 10.1136/adc.2009.177592

[18] Kloosterboer SM de Winter BCM Bahmany S . Dried blood spot analysis for therapeutic drug monitoring of antipsychotics: drawbacks of its clinical application: erratum. Ther Drug Monit. 2019;41:772. 31725696 10.1097/FTD.0000000000000712

[19] De Onis M. WHO Child Growth Standards: Length/Height-For-Age, Weight-For-Age, Weight-For-Length, Weight-For-Height and Body Mass Index-For-Age: Methods and Development. Geneva, Switzerland: World Health Organization; 2006.

[20] Aman MG Singh NN Stewart AW . The aberrant behavior checklist: a behavior rating scale for the assessment of treatment effects. Am J Ment Defic. 1985;89:485–491. 3993694

[21] Bravo Oro A Navarro-Calvillo ME Esmer C. Autistic behavior checklist (ABC) and its applications. In: Patel VB Preedy VR Martin CR, eds Comprehensive Guide to Autism. New York, NY: Springer New York; 2014:2787–2798.

[22] Turner RB Kojiro K Shephard EA . Review and validation of bayesian dose-optimizing software and equations for calculation of the vancomycin area under the curve in critically ill patients. Pharmacotherapy. 2018;38:1174–1183. 30362592 10.1002/phar.2191

[23] Hiemke C Bergemann N Clement HW . Consensus guidelines for therapeutic drug monitoring in neuropsychopharmacology: update 2017. Pharmacopsychiatry. 2018;51:e1–e62. 29390205 10.1055/s-0037-1600991

[24] Klampfl K Taurines R Preuss A . Serum concentrations, therapeutic response and side effects in children and adolescents with impulsive-aggressive symptoms during risperidone therapy. Pharmacopsychiatry. 2010;43:58–65. 20336598 10.1055/s-0029-1239540

[25] Taurines R Fekete S Preuss-Wiedenhoff A . Therapeutic drug monitoring in children and adolescents with schizophrenia and other psychotic disorders using risperidone. J Neural Transm (Vienna). 2022;129:689–701. 35303169 10.1007/s00702-022-02485-6 PMC9188514

[26] van der Esch CCL Kloosterboer SM van der Ende J . Risk factors and pattern of weight gain in youths using antipsychotic drugs. Eur Child Adolesc Psychiatry. 2021;30:1263–1271. 32839872 10.1007/s00787-020-01614-4 PMC8310848

[27] Hermans RA Ringeling LT Liang K . The effect of therapeutic drug monitoring of risperidone and aripiprazole on weight gain in children and adolescents: the SPACe 2: STAR (trial) protocol of an international multicentre randomised controlled trial. BMC Psychiatry. 2022;22:814. 36539734 10.1186/s12888-022-04445-6 PMC9769061

